# Regulation of CCL2 and CCL3 expression in human brain endothelial cells by cytokines and lipopolysaccharide

**DOI:** 10.1186/1742-2094-7-1

**Published:** 2010-01-04

**Authors:** Ray Chui, Katerina Dorovini-Zis

**Affiliations:** 1 Division of Neuropathology, Department of Pathology and Laboratory Medicine, Vancouver General Hospital and the University of British Columbia, Vancouver, BC, Canada

## Abstract

**Background:**

Chemokines are emerging as important mediators of CNS inflammation capable of activating leukocyte integrins and directing the migration of leukocyte subsets to sites of antigenic challenge. In this study we investigated the expression, release and binding of CCL2 (MCP-1) and CCL3 (MIP-1α) in an in vitro model of the human blood-brain barrier.

**Methods:**

The kinetics of expression and cytokine upregulation and release of the β-chemokines CCL2 and CCL3 were studied by immunocytochemistry and enzyme-linked immunosorbent assay in primary cultures of human brain microvessel endothelial cells (HBMEC). In addition, the differential binding of these chemokines to the basal and apical endothelial cell surfaces was assessed by immunoelectron microscopy.

**Results:**

Untreated HBMEC synthesize and release low levels of CCL2. CCL3 is minimally expressed, but not released by resting HBMEC. Treatment with TNF-α, IL-1β, LPS and a combination of TNF-α and IFN-γ, but not IFN-γ alone, significantly upregulated the expression and release of both chemokines in a time-dependent manner. The released CCL2 and CCL3 bound to the apical and basal endothelial surfaces, respectively. This distribution was reversed in cytokine-activated HBMEC resulting in a predominantly basal localization of CCL2 and apical distribution of CCL3.

**Conclusions:**

Since cerebral endothelial cells are the first resident CNS cells to contact circulating leukocytes, expression, release and presentation of CCL2 and CCL3 on cerebral endothelium suggests an important role for these chemokines in regulating the trafficking of inflammatory cells across the BBB in CNS inflammation.

## Background

The microenvironment of the brain is tightly regulated by the blood-brain barrier (BBB) the anatomical basis of which is the cerebral endothelium. The BBB endothelium is highly specialized and different morphologically, functionally and immunologically from small and large vessel EC of other organs. Under normal physiological conditions, the presence of interendothelial tight junctions and absence of a vesicular transport system restrict the entry of proteins, ions, lipid insoluble non-electrolytes and circulating haematogenous cells into the brain [1,2]. Yet in response to infectious, inflammatory diseases, ischemia, hemorrhage or trauma, there is an influx of leukocytes to sites of brain damage. Interactions between endothelial cells (EC) and circulating leukocytes have been increasingly implicated in the initiation and evolution of inflammatory processes in the central nervous system (CNS). Thus, molecular changes induced on the endothelium by cytokines lead to specific interactions with inflammatory cells that mediate their entry into the brain and accumulation at sites of antigenic challenge [3].

Chemokines are a family of chemoattractant cytokines characterized by their unique ability to both recruit and activate a variety of cell types. Currently, there are about fifty known chemokine members [4] which are divided into four sub-families by virtue of highly conserved N-terminal cysteine motifs (disulfide bonds) and the presence or absence of intervening amino acids. There are two major sub-families, which consist of the α- (or CXC) and β- (or CC) chemokines; and two minor sub-families, the γ- (or C) and δ-chemokines (or CX_3_C). Chemokines have been associated with a broad range of biological and pathological processes [5-7], which include angiogenesis [8], CNS development [9,10], atherosclerosis [11], cancer biology [12-14], autoimmune diseases [15], nervous system inflammation [16], asthma [17], and haematopoiesis [18].

There is considerable evidence that the β-chemokines CCL2 (MCP-1) and CCL3 (MIP-1α) play an important role in CNS inflammation. Several studies have shown the presence of CCL2 and/or CCL3 in multiple sclerosis (MS) lesions. In acute MS lesions, CCL2, CCL3 and CCL4 are selectively expressed by astrocytes, macrophages, and microglia in the lesion centre and in the surrounding white matter, whereas in actively demyelinating plaques CCL5 localizes on EC, perivascular cells and reactive astrocytes [19]. In acute and chronic active MS lesions, CCL2, along with CCL7 and CCL8, are expressed primarily by hypertrophic astrocytes and variably by inflammatory cells [20,21]. Significantly lower CSF levels of CCL2 have been reported in MS patients with active disease compared to controls and patients with stable MS [22,23]. In this regard, it has been shown that CCL2 is "consumed" by T lymphocytes and monocytes as they migrate across brain EC monolayers in vitro, leading to downregulation of the CCR2 receptor in response to CCL2, which may account for the decreased CSF CCL2 levels [24]. The expression of CCL3 has been associated with microglia and macrophages in the white matter lesions of MS patients [25].

In experimental allergic encephalomyelitis (EAE), the animal model of MS, the onset of the disease coincides with the mRNA expression of CCL2, CCL3 and other chemokines [26,27] and the accumulation of CXCL10 and CCL2 [28]. In the Lewis rat model, CCL2 mRNA increased before the onset of clinical signs, peaked with height of clinical disease, and declined with resolution [29]. CCL2 expression was localized on lymphocytes, macrophages, astrocytes, and EC and correlated with disease activity [30]. In chronic relapsing EAE, increased expression of CCL2, CXCL10 and KC was observed in astrocytes, whereas infiltrating leukocytes were the source of CCL3 and CCL5 [31]. In the SJL/J mouse, the expression of CCL3 correlates with the clinical onset of EAE and administration of anti-CCL3 Abs prevents the development of both acute and relapsing disease and infiltration of mononuclear cells into the CNS initiated by the transfer of neuroantigen peptide-activated T cells [32]. Furthermore, these chemokines could drive Th1/Th2 lymphocyte differentiation [33]. Vaccination of Lewis rats with naked DNA encoding CCL2 and CCL3 prevented EAE [34]. These data suggest that CCL2 and CCL3 have critical and non-redundant roles in the pathogenesis of autoimmune CNS inflammation.

In this study, we investigated the kinetics of expression and release of CCL2 and CCL3 by resting and cytokine or lipopolysaccharide (LPS) treated human brain microvessel EC using a well-characterized in vitro model of the BBB. We show that the constitutive expression and release of CCL2 by HBMEC is significantly upregulated following EC activation in a time-dependent manner. In contrast, CCL3 expression under resting conditions is negligible and its release requires stimulation by cytokines or LPS. Since leukocyte integrin activation by chemokines is required for firm adhesion and transendothelial migration, these findings further support the active participation of the BBB endothelium in neuroinflammation.

## Methods

### Endothelial cell cultures

#### Human brain microvessel endothelial cells (HBMEC)

HBMEC were isolated from normal brains at autopsy less than 12 hours post mortem by methods previously described [35]. The study has complied with all institutional policies and was approved by the ethics committees of the University of British Columbia and the Vancouver General Hospital. For these studies we used HBMEC isolated from five individuals ranging in age from 30 to 57 years. The cause of death included acute myocardial infarction (2) and motor vehicle accidents (3) without CNS involvement. Review of the clinical records ensured the absence of any pre-existing neurological, psychiatric, inflammatory disease or cancer. The freshly isolated clumps of HBMEC were seeded onto fibronectin-coated 96 well plates or 100 mm diameter plastic dishes (Corning Costar Corp., Cambridge, MA) and maintained in culture in complete media consisting of M199 supplemented with 10% horse plasma derived serum (Hyclone Laboratories, Logan, UT), 100 μg/ml heparin, 20 μg/ml endothelial cell growth supplement, 300 μg/ml glutamine (all from Sigma Chemical Co., St. Louis, MO) and 1% antibiotic/antimycotic solution (Life Technologies Inc.). The cells were maintained at 37°C in a humidified 5% CO_2_/95% air incubator and culture media were changed every other day. Endothelial cells reached confluence 7 to 10 days after plating. The endothelial origin and purity of the cells was determined by their strongly positive perinuclear staining for FVIIIR:Ag and binding of Ulex europeaus I lectin. Primary cultures from a single isolation were used for each experiment.

#### Antibodies and cytokines

For immunocytochemistry, the following primary antibodies (Abs) were used: monoclonal mouse anti-human CCL2 (10 - 40 μg/ml) and CCL3 (10 - 50 μg/ml) were purchased from Pepro Tech (Rocky Hill, NJ) and Chemicon International Inc. (Temecula, CA); anti-Factor VIII related Ag and Ulex europeaus Ab were both purchased from Dako Diagnostics Canada Inc (Mississauga ON). Secondary Abs used for these studies included the following: 5 nm gold-conjugated goat anti-mouse IgG (1:40) (Auroprobe LM GAM IgG) and 10 nm gold-conjugated goat anti-mouse IgG (1:20) (Auroprobe EM GAR IgG, Cedarlane Laboratories Ltd, Hornby, ON). Mouse anti-human follicle stimulating hormone (FSH) (BioGenex Laboratories, San Ramon, CA) was used as irrelevant isotype-matched control Ab. Recombinant human tumour necrosis factor α (TNF-α) and lipopolysaccharide (LPS) were obtained from Sigma Chemical Co. Recombinant human interferon-γ (IFN-γ) was obtained from the NIH AIDS Research and Reference Program. Recombinant human interleukin-1β (IL-1β) was obtained from Inter Medico (Markham, ON). The concentrations of cytokines and LPS used were based on our previous studies and are within the range of concentrations reported in the blood and CNS in various inflammatory and infectious diseases.

#### Induction of chemokine expression by cytokines and LPS

HBMEC monolayers were grown to confluence in replicate wells and incubated with cytokines for 24, 48 and 72 hrs. The cytokines used were TNF-α (10 - 100 U/ml), IFN-γ (200 - 500 U/ml), IL-1β (10 U/ml), bacterial LPS (5 μg/ml) and combinations of these cytokines. Supernatants of cytokine-treated monolayers were collected following the respective time points for detection of chemokine release by sandwich ELISA.

#### Reverse transcription PCR (RT-PCR)

Confluent HBMEC cultures grown on 100 mm diameter fibronectin- coated plates were used untreated or following 24 hr incubation with TNF-α (100 U/ml) and IFN-γ (500 U/ml). The cells of both unstimulated and stimulated cultures were removed using a rubber policeman, centrifuged and the cell pellets were frozen at -70°C.

Trizol (Life Technologies Inc.) was added to the frozen pellet, sonicated and the RNA extracted using chloroform. The RNA preparation was cleaned up using an RNeasy kit (Qiagen Inc., Mississauga, ON), according to the manufacturer's instructions. 2 - 5 μg of total RNA was reverse transcribed for 90 min at 37°C using Moloney Murine Leukaemia Virus reverse transcriptase (MMLV-RT, USB Corp., Cleveland, OH) and random hexamer primers (Amersham Pharmacia Biotech Inc., Piscataway, NJ). PCR was performed using 2.5 U/25 μl reaction AmpliTaq Gold (Applied Biosystems, Foster City, CA) with primers purchased from Life Technologies Inc. (Table 1) on 1 - 5 μl of cDNA under the following conditions: one initial cycle with dissociation at 94°C for 8 min, annealing at 55 - 60°C for 30 sec, extension at 72°C for 3 min; then cycling at 94°C for 1 min, 55 - 60°C for 30 sec and 72°C for 45 sec on an Applied Biosystems GeneAmp PCR System 9700 thermalcycler. GAPDH was used as an internal standard. The following were used as positive controls: pBluescript-hCCL2 (kind gift from Dr. Teizo Yoshimura, NCI/NIH, MD) and pBR322-hCCL3 (kind gift from Dr. Donald Forsdyke, Queen's University, Kingston, ON). PCR products were run on either a 6% polyacrylamide/TBE gel or a 2% agarose/TBE gel containing ethidium bromide (EtBr) and visualized under UV light. The RT-PCR experiments were repeated three times for each chemokine using different primary HBMEC cultures.

**Table 1:** RT-PCR primer sequences

GENE	PRIMER SEQUENCE
CCL2	*F: atg aaa gtc tct gcc gcc ctt ctg t †R: agt ctt cgg agt ttg ggt ttg ctt g
	
CCL3	F: atg cag gtc tcc act gct gcc ctt R: gca ctc agc tcc agg tcg ctg aca t
	
GAPDH	F: cca tgt tcg tca tgg gtg tga acc a R: gcc agt aga ggc agg gat gat gtt c

PCR primer sequences for CCL2 and CCL3 amplify fragments that are 298 and 274 base pairs in length, respectively. *F-forward/sense primer. †R-reverse/anti-sense primer.

#### Light microscopic intracellular localization of CCL2 and CCL3

Resting and cytokine treated (TNF-α [10-100 U/ml] and IFN-γ [200-500 U/ml]) confluent monolayers cultivated in triplicate wells of 96 well plates were washed with phosphate-buffered saline (PBS) supplemented with 1% bovine serum albumin (BSA, Sigma Chemical Co.) and 1% normal goat serum (NGS), and then fixed- permeabilized using buffered formaldehyde/acetone containing 0.03% Triton X-100 for 10 minutes. The cultures were then incubated with anti-CCL2 or anti-CCL3 monoclonal Abs diluted at 10 μg/ml in carrier buffer consisting of PBS containing 5% BSA and 4% NGS. At the end of the incubation period, cultures were washed and incubated with 5 nm gold-conjugated secondary Ab at 1:40 dilution for 1 hr. Following washing in wash buffer, the cultures were incubated with silver enhancement solution (Amersham Life Sciences, Buckinghamshire, England) and silver deposition was monitored for 22 - 26 min. Nuclei were counterstained with Giemsa. Controls included unstimulated HBMEC and cytokine treated cultures incubated with carrier buffer, isotype-matched irrelevant Ab (anti-human FSH) or normal mouse IgG (Cedarlane) instead of primary Abs. The cells were examined under a Nikon Labophot light microscope.

#### Immunoelectron microscopy

Confluent unstimulated HBMEC cultures and cultures treated with TNF-α (10 - 100 U/ml) and IFN-γ (200 - 500 U/ml) for 24 hrs were washed with PBS containing 1% BSA and 20 mM NaN_3 _and incubated with anti-CCL2 or CCL3 monoclonal Abs at 10 μg/ml in PBS containing 5% BSA, 4% NGS and 20 mM sodium azide (NaN_3_) for 30 min at room temperature. Following washing in wash buffer, the monolayers were incubated for 1 hr with the secondary Ab conjugated to 10 nm gold particles at 1:40 dilution in carrier buffer. Cells were then washed and fixed with cold 1/2-strength Karnovsky's fixative (2.5% glutaraldehyde and 2% paraformaldehyde in 0.2 M sodium cacodylate buffer) for one hr at 4°C. Following fixation, the cells were washed with 0.2 M sodium cacodylate buffer and post-fixed with 1% osmium tetroxide for 1 hr at 4°C. After block staining with uranyl magnesium acetate overnight at 4°C, the cultures were washed with sodium acetate buffer, dehydrated and embedded in Epon-Araldite. Longitudinal strips cut from the embedded cultures after curing of the plastic were re-embedded in Araldite for cross sectioning. Thin sections were examined using a Zeiss EM 910 electron microscope. One hundred cells from each resting or treated cultures were photographed under 25,000× magnification and the number of gold particles bound per μm of cell membrane on the apical, basal cell surface or subendothelial matrix was quantified, taking the magnification into account.

#### Enzyme-linked immunosorbent assay (ELISA)

HBMEC grown to confluence in triplicate wells of 96 well plates were used untreated or following incubation with various cytokines or LPS for 24 - 72 hrs. The supernatants were collected and analyzed by sandwich ELISA (Quantikine ELISA Kits, R & D Systems, Minneapolis, MN), as per manufacturer's instructions. Briefly, culture supernatants and provided standards were added to microtiter plates coated with the appropriate capture Ab. The application of detection Ab, along with substrate (TMB), allowed color development, which was stopped with 2N sulfuric acid. Absorbance was read with an ELISA microtiter plate reader at a wavelength of 490 nm. The assay sensitivity was <5 pg/ml for CCL2 and <10 pg/ml for CCL3. The generation of a standard curve using chemokine standards allowed the calculation of the quantity of chemokines released by HBMEC in the culture supernatants.

### Statistical analysis

Values derived from ELISA experiments were analyzed using analysis of variance (ANOVA) to determine significant differences between treatments. Student's t tests were used where differences were found. The Tukey test was also used as a multiple comparison method. Data that were not normally distributed were subjected to Kruskal-Wallis ANOVA on Ranks and Mann-Whitney U-tests. The chi-square test was used to analyze the immunoelectron microscopy data. Significant differences between cytokine treated cells and control groups are shown as (*) where p < 0.05.

## Results

### Detection of CCL2 and CCL3 RNA by RT-PCR

**Figure 1:**
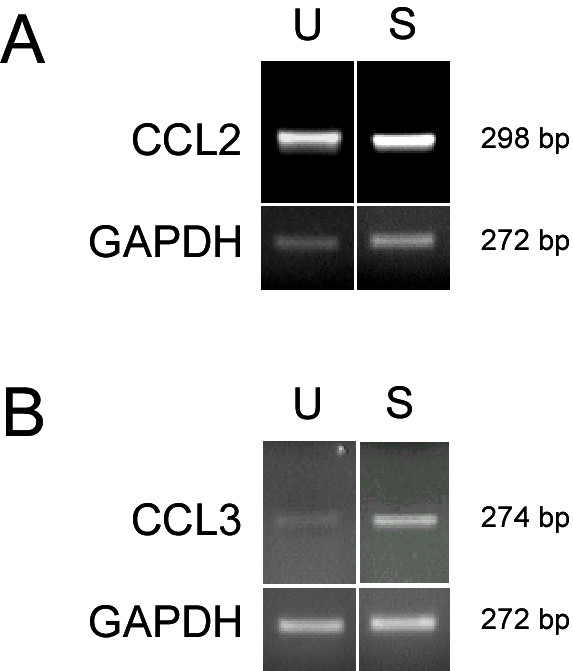
**RNA expression of CCL2 (A) and CCL3 (B) by HBMEC determined by semi-quantitative RT-PCR**. Increasing amounts of RNA (not shown) were used to detect differential expression in unstimulated HBMEC and cells treated with 100 U/ml TNF-α and 500 U/ml IFN-γ for 24 hrs. After amplification with gene-specific primers, CCL2 or CCL3 and GAPDH PCR samples were run on a 2% agarose gel after 35 and 25 cycles respectively, under the following conditions: pre-PCR step (94°C for 8 min, 55 - 60°C for 30 sec and 72°C for 3 min) and cycling (94°C for 1 min, 55 - 60°C for 30 sec and 72°C for 45 sec). The data shown are from one of three experiments for each chemokine with similar results.

RNA extracted from confluent monolayers of HBMEC with and without treatment with 100 U/ml TNF-α and 500 U/ml IFN-γ for 24 hrs was used to study the expression patterns of CCL2 and CCL3 in these cultures. The CCL2 and CCL3 gene-specific primers (Table 1) used for these studies amplified 298 and 274 base pair fragments, respectively. The expression of CCL2 RNA from both unstimulated and stimulated HBMEC was similar (Fig. 1A). There appeared to be no detectable upregulation of RNA expression following cytokine treatment. CCL2 cDNA cloned into pBluescript was used as the positive control in these experiments.

CCL3 RNA was found in barely discernible levels in unstimulated HBMEC (Fig. 1B). Incubation with 100 U/ml TNF-α and 500 U/ml IFN-γ for 24 hrs resulted in increase in RNA expression. The control used for these experiments was CCL3 cDNA cloned into a pBluescript vector.

### Immunocytochemistry

**Figure 2:**
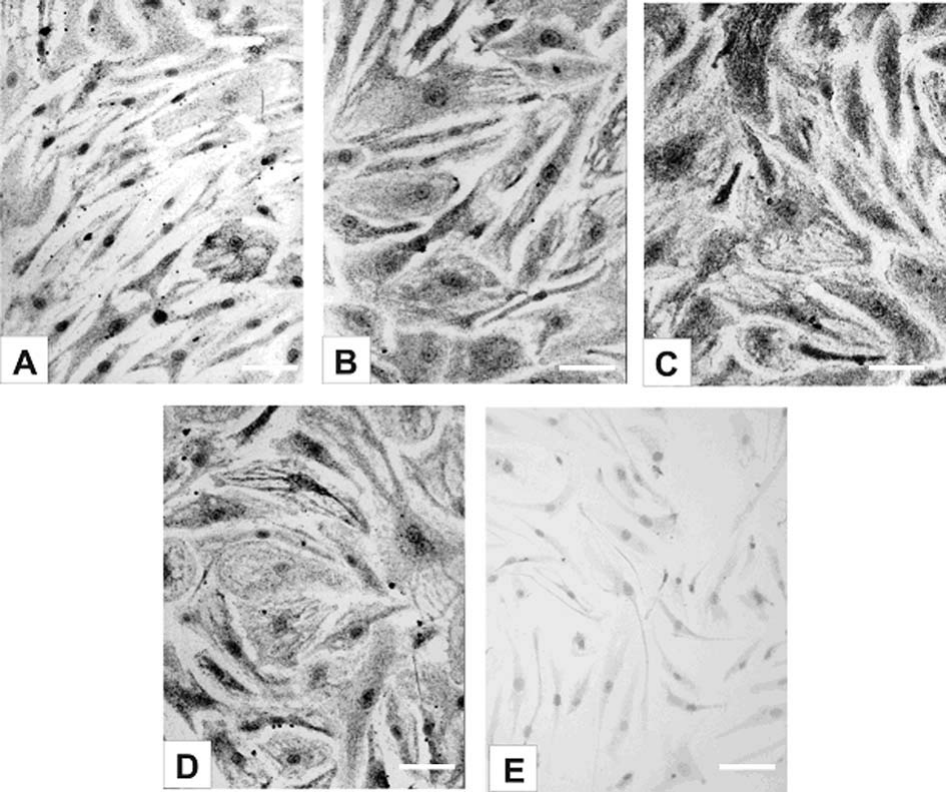
**Intracellular localization of CCL2 in HBMEC by immunogold silver staining**. (A) Unstimulated HBMEC constitutively express CCL2 as shown by the positive cytoplasmic staining in the form of fine, granular black deposits of silver-enhanced gold particles. Nuclei are counterstained with Giemsa. (B)-(D) The intensity of staining is markedly increased following incubation with (B) TNF-α (10 U/ml) for 48 hrs, (C) IL-1β (10 U/ml) for 72 hrs and (D) LPS (5 μg/ml) for 48 hrs. (E) Control cultures incubated with secondary antibody only show no staining. Scale bars = 50 μm.

**Figure 3:**
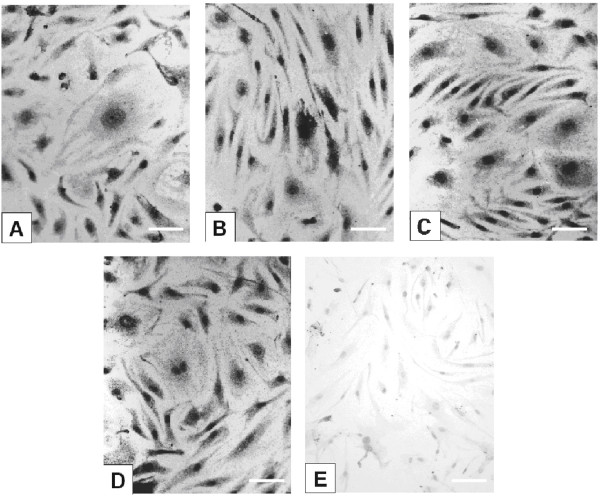
**Intracellular localization of CCL3 in HBMEC by immunogold silver staining**. (A) In untreated cultures, cytoplasmic staining is faint indicating minimal constitutive expression. Nuclei are counterstained with Giemsa. (B)-(D) The density of the granular black cytoplasmic deposits is increased in cultures treated with (B) TNF-α (100 U/ml)+ IFN-γ (200 U/ml) for 72 hrs, (C) IL-1β (10 U/ml) for 72 hrs and (D) LPS (5 μg/ml) for 48 hrs. (E) Control cultures incubated with secondary antibody only show no staining. Scale bars = 50 μm.

Immunogold silver staining was used to demonstrate intracellular CCL2 and CCL3 protein expression in confluent monolayers of unstimulated HBMEC and in cultures treated for 24 to 72 hrs with TNF-α, IFN-γ, IL-1β and LPS, or combinations of TNF-α and IFN-γ. All untreated cells exhibited positive staining for CCL2 in the form of fine, black, granular cytoplasmic deposits (Fig. 2A) indicating constitutive protein expression consistent with the constitutive RNA expression by RT-PCR. Treatment with cytokines or LPS resulted in increased diffuse cytoplasmic staining in the majority of cells, as compared to the untreated ones (Figs. 2B-D). Some variation in the intensity of staining between individual cells in both resting and stimulated cultures was usually present (Figs. 2A-D), however, no differences were detected between HBMEC from different donors. Unstimulated HBMEC monolayers showed uniformly slight cytoplasmic staining for CCL3 indicating minimal protein expression at all time points investigated (Fig. 3A) consistent with the low RNA expression by RT-PCR. Incubation with TNF-α, IFN-γ, IL-1β and LPS, or combination of TNF-α and IFN-γ resulted in increased intensity of cytoplasmic staining in all cells, with some variation between EC of the same culture (Figs. 3B-D), but not between different donor endothelial cells.

### Release of CCL2 and CCL3 by HBMEC in culture

**Figure 4:**
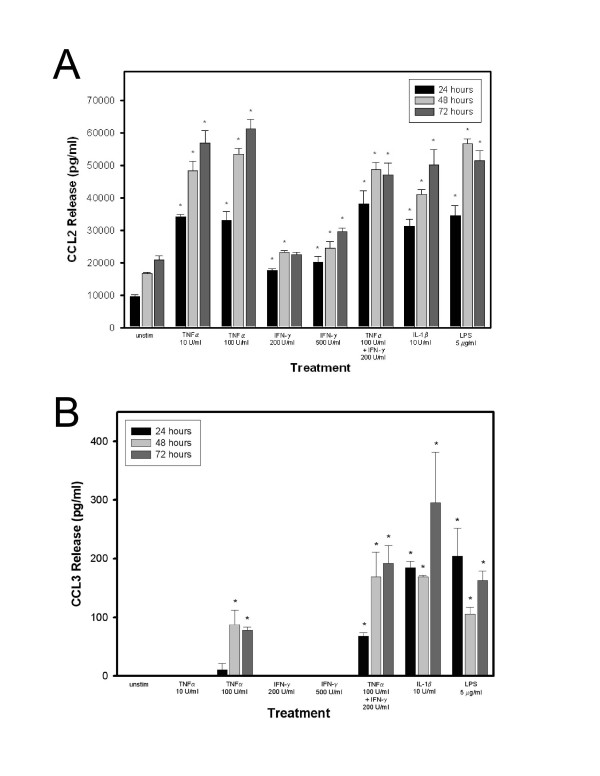
**Quantitation of CCL2 and CCL3 release by resting and cytokine stimulated HBMEC by ELISA**. (A) Detection of CCL2 protein in supernatants of HBMEC cultures under resting conditions and following incubation with cytokines and LPS. Chemokine release was determined by sandwich ELISA at the indicated time intervals. Values represent mean release (pg/ml) ± SEM (n = 3). Tukey test p ≤ 0.001; * p < 0.05 as compared to unstimulated HBMEC. Values shown represent one of two independent, representative experiments. (B) Detection of CCL3 protein in supernatants of resting HBMEC cultures and monolayers treated with cytokines and LPS. Chemokine release was determined by sandwich ELISA at the indicated time intervals. Values represent mean release (pg/ml) ± SEM (n = 3). ANOVA p ≤ 0.001; * p < 0.05 as compared to unstimulated HBMEC. Values shown are the results of one of two independent, representative experiments.

In order to determine the amount of CCL2 and CCL3 released by confluent HBMEC monolayers treated with TNF-α, IFN-γ, IL-1β, LPS, or combination of TNF-α and IFN-γ, culture supernatants from both resting and stimulated cultures grown in triplicate wells were removed at 24, 48 and 72 hrs post stimulation and analyzed by sandwich ELISA. Unstimulated HBMEC released 10 ~21 ng/ml of CCL2 over 24 - 72 hrs with significant increase in release following cytokine treatment in a time-dependent fashion (Fig. 4A). Treatment with TNF-α at 10 or 100 U/ml increased CCL2 release up to ~35 ng/ml at 24 hrs and up to a maximum of ~63 ng/ml at 72 hrs. This increase in CCL2 release was time-, but not concentration-dependent. Incubation with LPS (5 μg/ml) or IL-1β (10 U/ml) resulted in 124% and 156% increase in CCL2 release over unstimulated cultures, respectively, in a time-, but not concentration- dependent manner. Incubation of HBMEC with IFN-γ alone resulted in a modest, but not significant upregulation of release that was similarly time- but not concentration- dependent. Co-incubation with TNF-α (100 U/ml) and IFN-γ (200 U/ml) augmented CCL2 release to levels comparable with TNF-α treatment alone.

The release of CCL3 by confluent monolayers of HBMEC was measured by ELISA in culture supernatants similarly collected at 24, 48 and 72 hr time points. Unstimulated cells did not release any detectable protein (Fig. 4B). Stimulation with 100 U/ml TNF-α resulted in small amounts of protein release within the first 24 hrs and up to 100 pg/ml over 48 and 72 hrs. Lower concentrations of TNF-α (10 U/ml), as well as IFN-γ at 200 and 500 U/ml, failed to induce CCL3 release into the media. However, co-incubation of HBMEC with TNF-α (100 U/ml) and IFN-γ (200 U/ml) significantly augmented CCL3 release to a maximum of 200 pg/ml (p < 0.005) in a time-dependent manner. Treatment of the monolayers with IL-1β (10 U/ml) or LPS (5 μg/ml) induced the release of up to 200 - 400 pg/ml of this chemokine. There were some quantitative differences in the constitutive and stimulated release of both chemokines between HBMEC from different donors; however, the pattern of chemokine release after cytokine or LPS treatment was similar in all experiments.

### Differential binding of CCL2 and CCL3 to the surface of HBMEC

**Figure 5:**
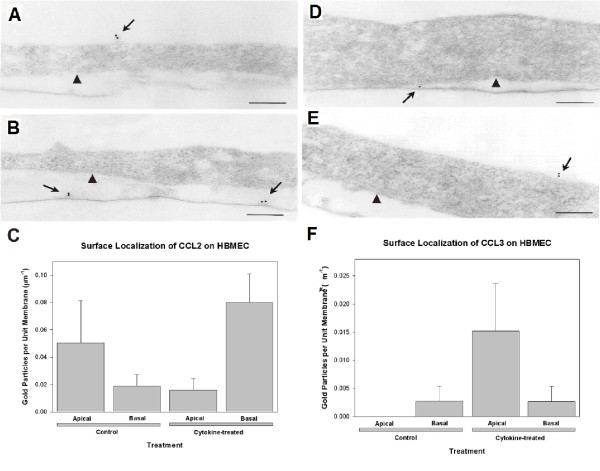
**Surface localization of CCL2 and CCL3 by immunoelectron microscopy**. (D, E) Surface localization of CCL2 on HBMEC by immunoelectron microscopy. (A) In resting monolayers, gold particles indicating the presence of CCL2 are bound mostly to the apical cell surface (arrow). (B) In cultures treated with TNF-α (100 U/ml) + IFN-γ (500 U/ml) for 24 hrs gold particles are preferentially bound to the subendothelial basal lamina-like material (arrows). Arrowheads in (A) and (B) indicate the basal cell surface. Scale bars = 0.25 μm. (C) Quantification of the number of gold particles bound to the apical and basal cell surface of unstimulated HBMEC shows no significant difference between apical and basal binding (p ≥ 0.1). In cytokine-treated HBMEC cultures, there is a significant increase in the number of gold particles bound to the basal cell surface and the basal lamina-like material versus the apical surface (p ≤ 0.01). The number of gold particles at the basal cell surface is significantly greater in activated versus resting HBMEC (p ≤ 0.01). (D, E) Surface localization of CCL3 on HBMEC by immunoelectron microscopy. (A) In resting monolayers occasional gold particles are bound to the basal cell surface only (arrow). (B) Following treatment with TNF-α (100 U/ml) + IFNγ (500 U/ml) for 24 hrs gold particles are preferentially bound to the apical surface (arrow). Arrowheads in (A) and (B) indicate the basal cell surface. Scale bars = 0.25 μm. (F) Quantification of the number of gold particles bound to the apical and basal cell surfaces of unstimulated and cytokine-treated HBMEC cultures shows no statistically significant differences between the different groups (p ≥ 0.15).

Chemokines are secreted molecules that exert their actions via binding to glycosaminoglycans at the endothelial surface and the extracellular matrix. In order to elucidate the binding patterns of CCL2 to the cell membrane, an immunoelectron microscopic approach was employed. An additional advantage of this method is the ability to visualize both the apical and basal binding of the chemokines. HBMEC cultures were used untreated or after stimulation for 24 hrs with cytokines (100 U/ml TNF-α + 500 U/ml IFN-γ). A small number of gold particles indicating the presence of CCL2 were associated with the cell surface of untreated HBMEC (Fig. 5A). Most of the gold particles were associated with the apical cell surface compared to the basolateral surface, although this difference did not reach significant difference (Fig. 5C). In cytokine-treated monolayers, CCL2 was redistributed and bound preferentially to the basal cell surface and the discontinuous, amorphous, basal lamina-like material underlying the basal surface compared to the apical one, as shown by the increased mean value (Figs. 5B, C). The number of gold particles bound to the basal cell surface was significantly greater in cytokine treated versus resting HBMEC (Fig. 5C). Gold particles were not observed along intercellular contacts. In contrast to CCL2, virtually no gold particles were identified on the apical cell surface, and only occasional gold particles, indicating the presence of CCL3, were bound to the basal cell surface of unstimulated HBMEC (Figs. 5D, F). Gold particles were not found along intercellular contacts. Upon cytokine treatment, CCL3 localized predominantly along the apical cell surface, whereas the number of gold particles associated with the basal cell surface remained the same. Thus, although binding to the basal surface was similar between untreated and treated HBMEC, the mean number of gold particles associated with the apical surface was greater in cytokine activated cells (Figs 5E, F).

## Discussion

The entry of inflammatory cells into the brain is a critical event in the pathogenesis of inflammatory and infectious diseases, as well as non-inflammatory conditions of the CNS, such as stroke and trauma. As an anatomical and immunological barrier, the BBB plays a central role in the recruitment of leukocytes in acute and chronic CNS inflammation. It is now well established that the transendothelial movement of leukocytes is a multi-step process, each step being mediated by specific interactions between EC adhesion molecules and their ligands on leukocytes [36]. Some of the molecular mechanisms involved in the trafficking of leukocytes across the BBB have been recently elucidated and involve membrane interactions between adhesion molecules induced on cerebral EC by inflammatory mediators and integrins or glycosylated ligands on leukocytes. Recent studies from this laboratory have shown that the adhesion and transendothelial migration of resting and activated T lymphocytes across the BBB depend upon the activation status of the endothelium and the T cells and are mediated by receptor-ligand interactions that are specific for each step and for each class of leukocytes [37,38].

In vivo and in vitro studies have established a critical role for chemokines in the leukocyte adhesion cascade. Binding of chemokines to their G-protein coupled receptors on leukocytes triggers inside-out signaling leading to rapid integrin activation and firm adhesion to the endothelium [39]. Although the expression of chemokines by glial cells in the CNS has been relatively well documented [21,40], human cerebrovascular chemokine expression has not been fully characterized. In the present study we show that CCL2 is constitutively expressed by HBMEC with marked increase of the intracellular protein and a 3-fold increase in the release of CCL2 into the media following treatment with TNF-α, IL-1β and LPS. Although IFN-γ alone had no effect on CCL2 release by HBMEC, combination of the highest concentrations of TNF-α and IFN-γ resulted in a 3-fold increase of the CCL2 levels in the supernatants.

Constitutive expression of CCL2 mRNA has been previously reported in porcine brain EC with upregulation upon stimulation with TNF-α [41]. In vitro studies using rat brain and retinal EC lines showed constitutive expression of CCL2 and increased release into the media following activation with TNF-α, IL-1β and IFN-γ [42]. Both the constitutive release at 2,000 - 2,700 pg/ml and the stimulated release at 5,500 pg/ml were much lower compared to HBMEC (up to 20,000 pg/ml and 60,000 pg/ml, respectively), which may reflect species differences in CCL2 expression and release. In extracerebral endothelium models, CCL2 RNA transcripts have been demonstrated in human aortic, pulmonary artery and umbilical vein endothelial cell (HUVEC) cultures, as well as in freshly removed human arteries and veins [43]. Expression of CCL2 mRNA has been detected in resting and cytokine activated HUVEC and human brain EC after IFN-γ stimulation [44]. Exposure of human cerebrovascular EC to hypoxic astrocyte-conditioned media for 4 - 8 hrs increased the release of CCL2 from a low constitutive level of 100 pg/ml to a modest 600 - 700 pg/ml, both significantly lower than the levels obtained in the present study [45]. This may be related to the short time of exposure to hypoxic media. Similarly, expression of CCL2 was induced in human brain-derived EC by endothelin-1 and ischemia [46]. A modest increase in CCL2 release, up to 1,800 pg/ml, has been reported in a human brain EC line after incubation with heat-killed Streptococcus suis [47]. The same strain had no effect on CCL2 expression by HUVEC. In addition to cytokines, injection of the HIV Tat1-72 protein into the mouse hippocampus was shown to increase the expression of CCL2 on brain vascular endothelium [48]. Substantial evidence indicates the importance of CCL2 in the induction and propagation of the inflammatory cascade. Thus, CCL2 was shown to stimulate T cell migration across microvascular endothelium [49] and to mediate the firm adhesion of monocytes under flow conditions [50]. In the CNS, knockout models for CCL2 and CCR2 provide strong evidence for the importance of the CCL2-CCR2 interaction [51,52]. Recent observations indicate that glia-derived CCL2 regulates the development of EAE by attracting TNF-α and iNOS-producing dendritic cells and macrophages to the CNS [53]. Furthermore, a recent study investigating potential mechanisms for HIV entry into the CNS indicates that CCL2 enhances the transmigration of HIV-infected leukocytes across the BBB via the upregulated expression of CCR2 [54].

In contrast to CCL2, the constitutive RNA and protein expression of CCL3 by HBMEC is negligible. Furthermore, resting HBMEC do not constitutively release CCL3 into the media. Treatment with TNF-α and IFN-γ increased RNA expression and incubation with individual cytokines or LPS upregulated protein expression and induced CCL3 release. However, under identical experimental conditions, the stimulated CCL2 release was typically two orders of magnitude greater than CCL3 release. IFN-γ alone has no effect on cerebral endothelial CCL3 expression, however, when combined with TNF-α, a synergistic effect resulted in higher protein levels compared to TNF-α alone. Expression of CCL3 by EC has been addressed in a limited number of studies. In animals, CCL3 has been reported in murine bone marrow EC [55] and in the endothelium of epineurial and endoneurial vessels following transection of the rat sciatic nerve [56]. Furthermore, expression of this chemokine was induced in a murine endothelial cell line by alloantigen-primed T cells [57]. In humans, endothelial expression of CCL3 has been documented in HUVEC incubated with activated platelets [58] or exposed to diamide [59] and LPS [60]. In addition, CCL3 has been localized to EC of blood vessels and splenic sinusoids in the hemophagocytic syndrome [61]. According to a recent report, transmigration of bone marrow-derived dendritic cells across mouse brain EC monolayers was increased in the presence of CCL3 concentration gradients [62]. The expression of CCL3 by cerebrovascular endothelium under resting and inflammatory conditions has not been previously addressed.

It is now well established that immobilization of chemokines by binding to glycosaminoglycans on the luminal EC surface enhances leukocyte adhesion, while binding to the abluminal surface and subendothelial matrix promotes their directional migration to sites of inflammation [63]. The present study shows that, following their release into the culture media, CCL2 and CCL3 bind to the surface of HBMEC and to the discontinuous basal lamina-like material under the basal cell surface in a polarized manner, which is distinct for each chemokine. Thus, under resting conditions, CCL2 binds preferentially to the apical (luminal) surface of HBMEC, whereas CCL3 shows minimal binding only to the basal (abluminal) EC surface, which is consistent with our protein synthesis and release results. In cytokine activated HBMEC, binding of CCL2 is redistributed towards the basal cell surface and that of CCL3 preferentially on the apical surface. These findings suggest that CCL3 may be primarily responsible for the initial recruitment and activation of CCR1 and/or CCR5 expressing cells, whereas CCL2 plays a greater role in establishing the chemotactic gradients necessary for the directional cell migration into the brain parenchyma. In accordance with these observations, previous studies have demonstrated the presence of specific and separate binding sites for CCL2 and CCL3 along the abluminal surface of human brain microvessels [64]. The differential binding of CCL2 and CCL3 to cerebral EC in an inflammatory milieu lends support to previous studies which, taking into account the CCL2 and CCL3 expression patterns and Ab therapy studies, suggest that CCL3 controls mononuclear cell accumulation during acute EAE, whereas CCL2 controls cellular infiltration during relapsing disease indicating that acute and relapsing EAE are regulated by the differential expression of CCL2 and CCL3 [65].

Previous in vitro studies from this laboratory have documented the expression and cytokine upregulation of the β-chemokines CCL4 and CCL5 by HBMEC [66] and their role in enhancing adhesion of memory and activated CD4+ T lymphocytes to cytokine treated HBMEC [67]. The present study points towards substantial differences in cytokine regulation of protein release among the four β-chemokines. The release of CCL2 and CCL3 is upregulated by TNF-α, IL-1β, LPS and TNF-α + IFN-γ, but not IFN-γ alone, in contrast to CCL5 which responds to TNF-α, IFN-γ and LPS, but not IL1-β. The release of CCL4 is augmented by LPS and combinations of TNF-α with IFN-γ or IL-1β, but not by single cytokine treatments. Overall, the release of CCL2 under resting and stimulated conditions is much greater than that of CCL3, CCL4 and CCL5. Additional differences exist in the distribution of bound chemokines to HBMEC. In resting monolayers, CCL2 and CCL5 are bound preferentially to the apical EC surface, CCL4 to both apical and basal surfaces and CCL3 only to the basal surface. Upon cytokine stimulation, this polarized expression is reversed for all four chemokines, with CCL2, CCL4 and CCL5 now present preferentially along the basal surface and subendothelial matrix, and CCL3 distributed mostly along the apical surface. These differences strongly suggest differential and possibly temporal roles of these chemokines in the regulation of leukocyte transendothelial migration in CNS inflammation.

## Conclusions

The studies reported herein demonstrate that brain microvessel EC synthesize CCL2 and CCL3 with significant upregulation after cytokine and LPS activation. The constitutive and stimulated production and release of CCL2 is quantitatively greater compared to CCL3. The polarized distribution of these chemokines on HBMEC under resting and simulated inflammatory conditions points towards possibly distinct functions, with CCL3 on the apical surface promoting leukocyte adhesion through integrin activation and CCL2 on the basal surface directing their migration across the BBB. These findings further emphasize the important role played by the cerebral microvascular endothelium in regulating inflammatory responses at the BBB.

## Competing interests

The authors declare that they have no competing interests.

## Authors' contributions

RC carried out the experiments and statistical analysis and contributed to the preparation of the manuscript. KD-Z conceived and designed the study and prepared the manuscript. Both authors have read and approved the final version of the manuscript.
